# Cost-Effectiveness of the Prenatal Detection of Congenital Heart Diseases: A Systematic Literature Review

**DOI:** 10.36469/001c.116147

**Published:** 2024-05-23

**Authors:** Darío Londoño Trujillo, Paula A. Castro García, Kristian K. Rojas López, Karen J. Moreno-Medina, María T. Dominguez Torres, Rodolfo J. Dennis Verano, Nestor F. Sandoval Reyes

**Affiliations:** 1 Fundación Santa Fe de Bogotá, Bogotá, Colombia; 2 Fundación Cardioinfantil-Instituto de Cardiología, Bogotá, Colombia

**Keywords:** fetal ultrasonography, systematic review, economic evaluation, prenatal diagnosis, congenital heart disease

## Abstract

**Background:** Congenital heart disease is the most common congenital condition worldwide, with a prevalence of 80 cases per 10 000 live births. In addition to perinatal morbidity and mortality, it entails long-term consequences such as multiple surgeries, prolonged hospitalizations, lifelong cardiac follow-up, reduced quality of life, risk of heart failure, and premature mortality in adulthood. This significant health and economic burden on healthcare systems and families highlights the relevance of evaluating the cost-effectiveness of methods for early detection of this condition. **Objective:** To conduct a systematic literature review (SLR) to identify and analyze existing economic evaluations on prenatal detection of congenital heart diseases through ultrasound, focusing on the reported cost-effectiveness results and the methodological quality of the evaluated studies according to established criteria. **Methods:** An SLR of economic evaluations was conducted following PRISMA guidelines. A quantitative synthesis of key methodological components of each economic evaluation was performed. The incremental medical costs, effectiveness measures, and cost-effectiveness ratios reported in each study were compiled and compared. The methodological quality was assessed according to compliance with the 24 CHEERS criteria. **Results:** We found 785 articles, of which only 7 met all inclusion criteria. Most were cost-effectiveness analyses, with the most common outcome being number of cases detected. Screening with only 4-chamber views interpreted by general practitioners or cardiologists were dominant strategies compared with screening with 4-chamber plus outflow views interpreted by a general practitioner. Fetal echocardiography was most effective but most expensive. Screening with 4-chamber and outflow view, followed by referral to a specialist, were recommended as the least expensive strategy per defect detected. On average, articles met 17 of the 24 CHEERS criteria. **Discussion:** While recent cost-effectiveness analyses demonstrated improved methodological quality, there was a lack of homogeneity due to differences in comparators and population subgroups analyzed. Despite this heterogeneity, fetal ultrasonography screening was consistently identified as a cost-effective strategy, with its cost-effectiveness heavily influenced by the expertise of the interpreting physician. **Conclusion:** Most studies recommend implementing obstetric ultrasonography screening, without routine fetal echocardiography, for detecting congenital heart diseases.

## INTRODUCTION

Congenital heart disease (CHD) comprises alterations in the shape and function of the heart, the circulatory system, and the great vessels, which can be detected during pregnancy or at birth, and occur during cardiac embryogenesis.^1,2^ CHD is the most common congenital condition worldwide, with a prevalence of 80 cases per 10 000 live births^3^ and is associated with significant perinatal morbidity and mortality.^4^ Although it is estimated that approximately 20% of the incidence of CHD can be attributed to genetic syndromes, exposure to teratogens, or maternal diabetes, the remaining 80% of cases occur in pregnant women without a family history of CHD or any other risk factor.^5^

Half of CHDs are serious malformations, which usually require surgical treatment during the first year of life and carry a higher risk of mortality, impaired neurological development, and a lower quality of life during childhood,^6^ as well as a significant economic burden on healthcare systems.^7^ However, the advances made in recent decades in the detection, management, and treatment of fetuses and newborns with these diseases have led to an improvement in their survival and quality of life, and a notable reduction in associated sequelae.

It is estimated that in Latin America 54 000 children are born with CHD each year, of which 41 000 require some type of treatment but only 17 000 undergo surgery.^8^ According to Tassinari et al,^1^ a prevalence of 15.1 per 10 000 live births was estimated, placing CHD as the second-leading cause of death in children under 1 year of age, with a mortality of 0.12 per 10 000 births.

Given the aforementioned, it becomes imperative to conduct comprehensive monitoring of maternal and fetal health throughout the entirety of the pregnancy. This entails regular health assessments, analytical evaluations, and scheduled fetal ultrasound examinations over the 40-week gestation period.^6^ Ultrasound and specific blood tests are standard components of routine prenatal care. These procedures are safe and often guide decisions regarding the necessity for advanced studies during pregnancy or at birth, and the need for emergency medical interventions postpartum.^9,10^ Improved screening and prenatal diagnosis techniques enhance healthcare preparedness by identifying candidates for surgical interventions, ultimately reducing mortality and morbidity associated with these conditions at birth. The aim of this systematic literature review (SLR) on economic evaluations is to identify and analyze existing economic evaluations on prenatal detection of CHD through ultrasound, focusing on the reported cost-effectiveness results and the methodological quality of the evaluated studies according to established criteria.

## METHODS

### Search

An SLR was carried out to assess the cost-effectiveness of diagnostic imaging in detecting CHD during pregnancy using a combined PRISMA^11^ and CHEERS approach.^12^

To carry out the SLR, the Center for Reviews and Dissemination (CRD) of the University of York was consulted and specialized information on economic evaluation and technology assessment reports was collected from different databases, including the Cochrane Library, MEDLINE, EMBASE, PSYCINFO, CINAHL, and others. In addition to the search in the CRD, EMBASE, OVID, INAHTA, LILACS, NICE, PubMed, TRIP Database, and Google Scholar were consulted. As many databases as possible were included to broaden the coverage of published articles, reduce search bias, and ensure completeness of the search. All searches were conducted in June 2021.

Once the databases were defined, a search protocol was structured for each based on questions structured in the Population, Intervention, Comparison, Outcome format. Terms related to economic evaluations were included in nonspecialized databases on the subject. The keywords used in all the databases were “cost-benefit analysis,” “heart defects congenital,” “machine learning,” “prenatal diagnosis,” and “prenatal ultrasonography.” Search protocols are shown in the **Supplementary Online Material** (**Appendix 1**).

Inclusion criteria were (1) complete economic evaluations comparing costs and health benefits with incremental analysis among alternatives, (2) targeting pregnant women undergoing prenatal diagnosis of congenital heart malformations and (3) evaluating ultrasound, ultrasonography, or magnetic resonance imaging as comparators. No filters were applied for publication date, language, or quality of information sources. Studies not meeting these criteria were excluded.

Based on the established inclusion criteria, the list of titles and abstracts identified from the search was reviewed. Articles identified as relevant were independently reviewed in full text by 3 randomly paired researchers (C.P., R.K., L.D.). In cases where the abstract did not provide sufficient information, the methodology and results were reviewed for the application of the criteria. Lack of consensus between the 2 raters was resolved by a third rater.

### Synthesis of Evidence

The articles evaluated as pertinent were summarized in a table with their most relevant characteristics, in accordance with the recommendations of the Cochrane Collaboration, which include among its important domains the population, type of economic evaluation, health effects, type of costs, source of outcomes, and results. In addition to these, information regarding the year of publication, country of origin, perspective adopted, time horizon and interventions evaluated were also incorporated.

### Compilation of Cost-Effectiveness Results

From the evidence synthesis, relevant cost-effectiveness data were extracted from the studies, such as the costs of the interventions, incremental cost, reported effectiveness, incremental effectiveness, incremental cost-effectiveness ratio (ICER), or net monetary benefit. The studies were grouped between those evaluating congenital malformations of any nature and those evaluating congenital heart malformations.

### Methodological Assessment of the Evidence

To assess the methodological quality of the economic evaluations, each selected article was evaluated according to the criteria established by CHEERS in its Spanish-language version.^12^ The CHEERS checklist contains 24 items, divided into 5 components: title and abstract, methods, results, discussion, and others. Two researchers (C.P., R.K.) independently evaluated these components. In the event of any discrepancy, a consensus was sought between them; if this was not achieved, a third evaluator (L.D.) was used to resolve the conflicting items. First, we determined how many of the 24 CHEERS criteria each study met and then assessed whether these criteria were incorporated into the text correctly, acceptably, questionably, or unacceptably (not included), according to each reviewer. In this context, it was established that a correct criterion corresponded to an explicit mention of the evaluated item with its due justification, while an acceptable criterion referred to those cases in which the criterion was mentioned explicitly but not in depth. A questionable criterion made no explicit mention of the item, but it could be deduced that it was applied based on other methodological components, while an unacceptable criterion was one which was not mentioned and from which it could not be deduced that it was considered. Zotero® was used as bibliographic manager.

## RESULTS

### Search Results and Narrative Synthesis of Evidence

**Figure 1.**
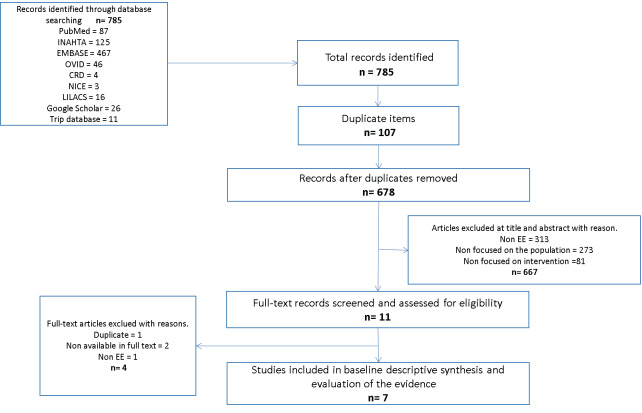
Flowchart of the Study Selection Process Abbreviation: EE, economic evaluation.

In all, 785 articles were identified, of which 103 were duplicates. The titles and abstracts of 682 were reviewed, and 667 were excluded for not meeting the inclusion criteria. Subsequently, 11 articles were selected for full-text reading. Among these, 4 were excluded for various reasons: they did not constitute an economic evaluation, were not available in full text, or were repeated texts published under different titles. Seven were included in the evidence synthesis and appraisal.^13–19^ The study selection process is presented through the flowchart proposed by the PRISMA^11^ statement in **Figure 1**.

Four articles were carried out from the perspective of the national health authority of their respective countries,^13,14,18,19^ 2 from the social perspective^15,17^ and 1 from the point of view of the service provider.^16^ Four did not report the time horizon in which costs and health consequences were evaluated,^13,16,17,19^ 2 adopted short-term horizons, taking as reference the time necessary for the diagnosis,^18^ and 1 with a time horizon 1 year after childbirth.^15^ Only 1 study performed the analysis with a vital horizon.^14^ In total, 4 cost-effectiveness analyses,^13,16,18,19^ 2 cost-utility analyses,^14,15^ and 1 cost-benefit analysis^17^ were found. The main characteristics of the studies are shown in **Table 1**.

**Table 1.** Main Characteristics of Articles

**Author**	**Title**	**Year**	**Country**	**Study Population**	**Perspective**	**Intervention**	**Comparison**	**Type of Economic Evaluation**	**Outcome Indicator**	**Source and Type of Cost**
Brown et al^19^	Choosing options for ultrasound screening in pregnancy and comparing cost effectiveness: a decision analysis approach	1998	UK	Pregnant women Evaluate lethal anomalies in the fetus	NHS	Routine US during 1st trimester	Do not perform US US and NT in the 1st trimester US in 2nd trimester US in 3rd trimester Different combinations between options	Cost-effectiveness	Detected anomalies	Direct medical costs Sources: Literature review and NHS reported rates
Vintzileos et al^17^	Routine second-trimester ultrasonography in the United States: a cost-benefit analysis	2000	USA	Pregnant women with low obstetric risk (fetus malformations)	Society	US in a tertiary-level center	US in primary- and secondary-level centers.	Cost-benefit	Avoided costs	Direct and indirect costs Source: Waitzman et al (1994 and 1998)
Vanara et al^13^	Economic evaluation of ultrasound screening options for structural fetal malformations	2004	Italy	Pregnant women between the 19th and 21st week Evaluate structural fetal malformations	Sanitary Authority	Structured gestational screening program with US to detect fetus malformations.	No structured screening program	Cost-effectiveness	Detected anomalies	Direct costs from NHS records Source: Vintzileos et al (2000)
Pinto et al^18^	Cost-effectiveness of prenatal screening strategies for congenital heart disease	2014	USA	Pregnant women with low obstetric risk Evaluate the presence of CHD in the fetus	Payer	US during the 2nd trimester with 4C view and subsequent referral to MFM, (4C MFM)	US during 2nd trimester with 4C and subsequent referral to a pediatric cardiologist (4C → card) US during 2nd trimester with 4C + outflow tracts, subsequent referral to MFM (4C + outflow → MFM). US during 2nd trimester with 4C + outflow tracts, subsequent referral to pediatric cardiologist (4C + outflow → card). US during 2nd trimester with 4C + outflow tracts performed by MFM, subsequent referral to pediatric cardiologist (MFM 4C + outflow → card) Stepped screening with NT in the 1st trimester, followed by US with view of outflow tracts in the 2nd trimester (NT + outflow) Universal fetal echocardiography	Cost effectiveness	CHD cases detected	Direct medical costs Source: 2012 National Medicare Rates
Mistry et al^14^	The cost-effectiveness of prenatal detection for congenital heart disease using telemedicine screening	2013	UK	Pregnant women with “standard risk” to evaluate the presence of CHD in the fetus	NHS	CHD screening with telemedicine	Usual in-person management	Cost utility	QALY	Direct costs Sources: Literature review and expert opinions
Chung et al^15^	The cost-effectiveness of prenatal congenital heart defect screening methods in In vitro fertilization (IVF) pregnancies	2020	USA	Women who got pregnant through IVF divided into 2 subgroups: IVF with conventional fertilization and IVF with ICSI	Society	Anatomical ultrasonography Selective fetal echocardiography after abnormal detailed anatomical study	Fetal echocardiography for all IVF pregnancies with ICSI Fetal echocardiography for all IVF pregnancies	Cost utility	QALY and CHD cases detected	Direct and indirect costs Sources: Literature review, Medicare, and Medicaid
Finneran et al^16^	The accuracy and cost-effectiveness of selective fetal echocardiography for the diagnosis of congenital heart disease in patients with pregestational diabetes stratified by hemoglobin A1c	2019	USA	Pregnant women with a diagnosis of pregestational diabetes	Not reported	US	Echocardiography for all patients with elevated A1c hemoglobin Selective echocardiography only after an abnormal US	Cost effectiveness	CHD cases detected	Direct medical cost Source: Medicare rates

Abbreviations: 4C, four-chamber; CHD, congenital heart disease; ICSI, intracytoplasmic sperm Injection; IVF, in vitro fertilization; MFM, maternal-fetal medicine; NHS, National Health Service; NT, nuchal translucency; US, ultrasound.

Regarding the studies’ country of origin, 4 were conducted in the United States,^15–18^ 2 in the United Kingdom,^14,19^ and 1 in Italy.^13^ These studies were published between 1998 and 2020. The target population focused on pregnant women at low obstetric risk who were evaluated for various structural malformations of the fetus^13,17,19^ including cardiac malformations, pregnant women at standard risk, low-risk or gestational diabetes,^13^ and in vitro fertilization,^15^ to evaluate the presence of CHD in the fetus.^14,15,18^

Studies focused on the detection of congenital anomalies^13,17,19^ took as an intervention the routine use of ultrasound, either in the first trimester of pregnancy,^19^ performed in a tertiary hospital center,^17^ or as part of a universal screening program.^13^ In these cases, the cost-effectiveness ratio of performing ultrasound at different times of pregnancy, in less complex hospital centers, or in selective strategies for its performance was evaluated. Articles that specifically addressed the detection of CHD evaluated the cost-effectiveness of fetal ultrasound compared with fetal echocardiography^15,16^ or with fetal ultrasound plus 4-chamber view and outflow tracts with subsequent referral to different clinical specialists for review.^15^ Mistry et al^14^ evaluated CHD screening using telemedicine, in which abnormal ultrasound images were reviewed by an expert, and compared it with usual management, in which it is not always possible that a specialist doctor can read the test results.

The most used outcome was correctly detected cases,^13,16,18,19^ whether they were heart disease or malformations, followed by quality-adjusted life-years^14,15^ and costs avoided in the case of cost-benefit analysis.^17^ Regarding the type of costs evaluated, direct medical costs were taken in all cases; in addition, Vintzileos and Chung included indirect or productivity costs in the analysis.^15,17^ Literature review was the usual source of effectiveness,^13,15,18,19^ although in some cases effectiveness came specifically from a secondary source such as the RADIUS clinical trial^17,20^ or from primary data collected in a timely manner for the economic evaluation.^14^

Given that the diagnostic processes address the short term, the type of model most used by the studies was the decision tree^13–15,18,19^; only 2 studies carried out statistical^16^ and cost^17^ analyzes on primary data. In most cases, only deterministic or scenario sensitivity analyzes were performed, accounting for the best and worst possible scenario with the available information. Only 2 articles^14,18^ incorporated probabilistic sensitivity analyzes into their results.

### Cost-Effectiveness

The reported costs varied depending on the outcome evaluated, that is, whether they only referred to heart disease detection or if other malformations were included. The nature of the intervention also influenced the cost, since in the case of universal screening programs, the costs tended to be higher than in those cases in which programs focused on a specific population group were evaluated. Finneran et al^16^ performed the evaluation in women with a diagnosis of gestational diabetes and reported that the additional cost of incorporating fetal echocardiography into those abnormal ultrasonographies would be USD $145 per patient, but would be USD $18 200 when evaluating the incremental cost-effectiveness ratio. In the case of implementing fetal echocardiography universally for all pregnant women with diabetes, the additional cost compared with ultrasonography would be USD $381, congruent with the results obtained by Pinto et al,^18^ who obtained similar incremental costs but with an incremental effectiveness of only 3 additional cases detected in a population of 1000 patients. Results of the incremental costs and effectiveness are shown in **Table 2**.

**Table 2.** Cost-Effectiveness Results

**Author**	**Interventions**	**Costs**	**Change in Costs**	**Effectiveness**	**Change in Effectiveness**	**ICER/Net Monetary Benefit**
**General evaluation of congenital malformations**						
Brown et al (1998)	Do not perform US		NR		NR	
	Routine US in 1st trimester	£30 450		0.53 cases detected		ICER = £57 453/case detected
	US + NT in 1st trimester	£30 583		1.58 cases detected		ICER = £19 356/case detected
	US during 2nd trimester	£31 139		5.85 cases detected		ICER = £5323/case detected
	US during 3rd trimester	£31 198		6.30 cases detected		ICER = £4952/case detected
Vintzileos et al (2000)	US in tertiary center	USD $270	NA	Avoided costs: USD 367−USD459	NA	USD 97−USD189 millions
	US in primary- and secondary-level centers	USD $270		Avoided costs: USD 109−USD201		(USD 69−USD161 millions)
Vanara et al (2004)	Structured gestational screening program with US	€400 536 721	-€104 469 663	7071 detected malformations	685 avoided malformations	ICER = 56 637/detected malformation
	No structured program	€505 006 384		6386 detected malformations		ICER = 79 085/detected malformation
**Specific evaluation of CHD**						
Pinto et al (2014)	4C + outflow → MFM	USD $169.33		3.53 detected cases		
	4C + outflow → card	USD $169.84	USD $0.51	4.42 detected cases	0.9	USD $579
	4C → MFM	USD $170.37	USD $1.04	2.37 detected cases	−1.16	Dominated
	4C → card	USD $171.76	USD $2.43	2.96 detected cases	−0.57	Dominated
	MFM 4C + outflow → card	USD $216.53	USD $47.20	5.07 detected cases	1.54	USD $30 591
	NT + outflow	USD $316.58	USD $147.25	5.06 detected cases	1.53	Extended dominance
	Fetal echocardiography	USD $513.72	USD $344.39	6.59 detected cases	3.06	USD $112 560
Mistry et al (2013)	Usual management	£12 906		23.24 QALY		
	Universal screening through telemedicine	£12 876		23.282 QALY		Dominant
Chung et al (2020)	Ultrasonography	USD $8119		1.74487 QALY		
	Echocardiography for ICSI only	USD $8408	USD $289	1.74497 QALY	0.0001	USD $2 890 000
	Echocardiography for all IVF	USD $8560	USD $441	1.74499 QALY	0.00012	US $5 692 457
Finneran et al (2019)	Detailed US	USD $195.13				
	Selective echocardiography (only in case of abnormal ultrasonography)	USD $340.29	USD $145.16		0.007936577	USD $18 290
	Universal echocardiography (all cases of elevated A1c)	USD $577.08	USD $381.95			USD $28 875

Abbreviations: 4C, four-chamber; CHD, congenital heart disease; ICER, incremental cost effectiveness ratio; ICSI, intracytoplasmic sperm Injection; IVF, in vitro fertilization; MFM, maternal-fetal medicine; NHS, National Health Service; NT, nuchal translucency; US, ultrasound; USD, US dollar.

### Compliance with Methodological Criteria

The mean number of CHEERS criteria met by the articles was 17. As expected, the articles published between 1998 and 2004 did not meet most of the quality criteria established by the instrument, obtaining a mean compliance with 13 criteria. By contrast, articles published after 2014 showed improvement in the methodological indicator, with an average compliance with 20.5 criteria.

**Figure 2.**
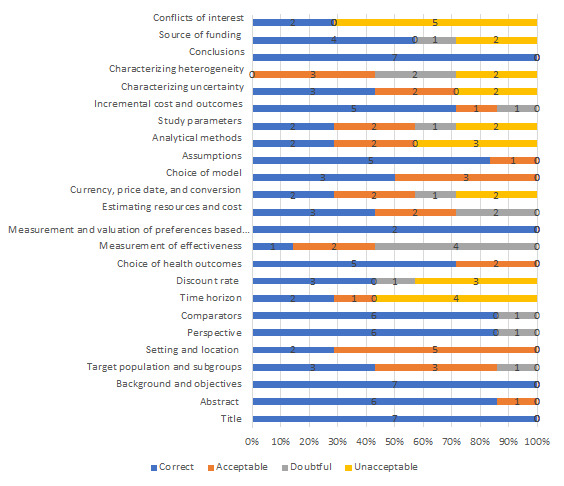
Levels of Compliance with CHEERS Methodological Criteria

In general, the weakest methodological criteria among the selected group of articles were obtaining measures of effectiveness, analytical methods, characterization of both uncertainty and heterogeneity, and declaration of conflicts of interest. The percentages of acceptability for these criteria among the set of articles are shown in **Figure 2**.

## DISCUSSION

While the cost-effectiveness studies identified varied in their methodological quality, with more recent studies (published within the last 10 years) generally adhering better to the CHEERS criteria, they reached different conclusions depending on the specific screening strategy evaluated. Nonetheless, a common trend emerged, recommending the implementation of fetal ultrasonography screening (FUS) for the detection of CHD. This has important clinical implications for early detection, management, and improved maternal and fetal health outcomes. Vintzileos et al^17^ found that performing FUS in the second and third trimester of pregnancy is a cost-effective strategy and is effective to detect congenital anomalies of the fetus if performed in a tertiary-care hospital. This idea is supported by Pinto et al,^18^ who suggest that the cost-effectiveness of second-trimester ultrasound for detecting CHD can be improved by adding assessment of the outflow tracts. It also suggests that the type of medical specialist interpreting the ultrasound results has a significant impact on the correct detection of cases and, consequently, on its cost-effectiveness, highlighting the implications for medical training and expertise in this area.

In addition to the 2 previous studies, Mistry et al^14^ found a relationship between the cost-effectiveness of FUS and medical expertise in its reading, indicating how the prenatal screening strategy for CHD, mediated by telemedicine, can be a cost-effective strategy. In the telemedicine screening program, FUS is analyzed by expert medical personnel, allowing for greater precision in the diagnosis.

The 2 studies that address specific populations—pregnant women with gestational diabetes^16^ and pregnancies due to in vitro fertilization^15^—agree that the routine use of fetal echocardiography in these populations is not a cost-effective strategy, since the additional cases of CHD that can be detected in relation to FUS are marginal and at a high cost. Both studies, and that of Pinto et al,^18^ recommend the use of echocardiography only in case of abnormal results on ultrasonography, which has economic implications in terms of resource allocation and accessibility to these services. There are few cost-effectiveness studies of FUS in the literature and even fewer methodologically well-designed studies, perhaps because there are still discrepancies about the usefulness of FUS for detecting heart disease in the first trimester, or because of doubts about the accuracy of interpretation of the results by nonspecialist medical personnel. This highlights the need for further research in this area, including the development of cost-effectiveness models tailored to local contexts and the evaluation of training strategies or incorporation of artificial intelligence (AI) technologies.

The variations in the measurements of the costs, the different years in which they were carried out, and the costs identified point to an important limitation of comparison among the different studies. Likewise, application of these results to the Colombian territory based on the cost-effectiveness ratio data obtained in other places, such as those described in this study, is not possible due to the great differences in costs and in other parameters. Therefore, it is essential to perform this type of analysis in the Colombian context. Undoubtedly, the aspects described in this paper highlight the need for cost-effectiveness studies in prenatal screening for congenital malformations to determine which mechanism may be the most clinically and economically efficient.

The recent emergence of new AI-assisted technologies has revolutionized the field of detecting congenital malformations and other diseases. Using advanced machine-learning algorithms, these tools can analyze medical images, such as ultrasound and MRI scans, providing diagnostic support to doctors regardless of their level of training. The incorporation of such technologies is a clinical and economic challenge, as it is necessary to ensure that their implementation will bring significant gains in patient health. Therefore, it is essential to establish the effectiveness and cost-effectiveness of the usual medical practice.

## CONCLUSIONS

Good-quality cost-effectiveness studies indicate that obstetric ultrasonography is a cost-effective strategy for detecting CHD, provided that the ultrasound results are interpreted by skilled and experienced professionals. Most of the studies recommend the implementation of ultrasonography, without routine echocardiography.

## Supplementary Material

Online Supplementary Material
