# Supplementary material for: Cost-Effectiveness of the Prenatal Detection of Congenital Heart Diseases: A Systematic Literature Review
Source: J Health Econ Outcomes Res. 2024 May 23;11(1):141–8. doi: 10.36469/001c.116147 (PMC11127739; doi:10.36469/001c.116147)
Supplement: Online Supplementary Material [file jheor_2024_11_1_116147_228775.pdf]

Journal of Health Economics and Outcomes Research

Online Supplementary Material

Cost-Effectiveness of Prenatal Detection of Congenital Heart Diseases: A Systematic Literature Review. *JHEOR*. 2024;11(1):141-148. doi:10.36469/jheor.2024.116147

PICS Question

Table S1: Keywords Used in the Search for Economic Evaluations

Search Protocols Used in Each Consulted Database

This supplementary material has been provided by the authors to give readers additional information about their work.

This is an open-access article distributed under the terms of the Creative Commons Attribution 4.0 International License (CCBY-4.0). View this license's legal deed at http://creativecommons.org/licenses/by/4.0 and legal code at http://creativecommons.org/licenses/by/4.0/legalcode for more information.

The initial search strategy aimed to retrieve evidence on the cost-effectiveness of incorporating artificial intelligence in the prenatal diagnosis of congenital heart disease (CHD) via fetal ultrasound. However, as the search results indicate, no relevant studies were identified. Consequently, the decision was made to conduct a search on the cost-effectiveness of routine prenatal detection of CHD using conventional ultrasound, regardless of the comparator intervention. The detailed search strategies employed across the consulted databases are presented below.

PICS QUESTION

- **Population:** Pregnant women between 18 and 23 weeks of pregnancy
- **Intervention:** Obstetric ultrasound assisted by machine learning
- **Comparison:** Conventional obstetric ultrasound
- **Study design:** Economic evaluation

Table S1. Keywords Used in the Search for Economic Evaluations

Population	Intervention	Comparison	Study Design
Pregnant women*	Machine learning*	Prenatal diagnosis*	Cost-benefit analysis*
Heart defects, congenital*	Artificial intelligence*	Prenatal screening	Cost-effectiveness analysis
CHD	Data mining*	Ultrasonography, prenatal*	Cost utility analysis
Fetal heart*	Automated diagnosis	Obstetric ultrasonography	Economic evaluation
Congenital heart defect		Prenatal ultrasound	
		Detailed anatomy ultrasound	
		Anatomy scan	
		Magnetic resonance imaging*	
		Imaging, 3-dimensional*	

Words marked with * correspond to MeSH terms.

Abbreviation: CHD, congenital heart disease.

SEARCH PROTOCOLS USED IN EACH CONSULTED DATABASE

PubMed (Search Date: June 12, 2021)

Search including intervention: ((pregnant women[MeSH Terms]) OR (Heart defects, Congenital[MeSH Terms]) OR (Congenital heart disease) OR (fetal heart[MeSH Terms]) OR (congenital heart defect)) AND ((machine learning[MeSH Terms]) OR (artificial intelligence[MeSH Terms]) OR (data mining[MeSH Terms]) OR (Automated diagnosis)) AND ((prenatal diagnosis[MeSH Terms]) OR (prenatal screening) OR (Ultrasonography, Prenatal[MeSH Terms]) OR (Obstetric ultrasonography) OR (Prenatal ultrasound) OR (Detailed Anatomy Ultrasound) OR (Anatomy scan) OR (Magnetic Resonance Imaging[MeSH Terms]) OR (Imaging, Three-Dimensional[MeSH Terms])) AND ((Cost-Benefit Analysis[MeSH Terms]) OR (Cost-Effectiveness Analysis) OR (Cost Utility Analysis) OR (Economic Evaluation))

Found papers = 0

Search excluding intervention: ((pregnant women[MeSH Terms]) OR (Heart defects, Congenital[MeSH Terms]) OR (Congenital heart disease) OR (fetal heart[MeSH Terms]) OR (congenital heart defect)) AND ((prenatal diagnosis[MeSH Terms]) OR (prenatal screening) OR (Ultrasonography, Prenatal[MeSH Terms]) OR (Obstetric ultrasonography) OR (Prenatal ultrasound) OR (Detailed Anatomy Ultrasound) OR (Anatomy scan) OR (Magnetic Resonance Imaging[MeSH Terms]) OR (Imaging, Three-Dimensional[MeSH Terms])) AND ((Cost-Benefit Analysis[MeSH Terms]) OR (Cost-Effectiveness Analysis) OR (Cost Utility Analysis) OR (Economic Evaluation))

Found papers = 87

EMBASE (Search Date: June 13, 2021)

Search including intervention: ('pregnant woman'/exp OR 'pregnant women' OR 'heart defects congenital diagnosis'/exp OR 'congenital heart disease'/exp OR 'fetus heart'/exp OR 'congenital heart malformation'/exp) AND ('machine learning'/exp OR 'artificial intelligence'/exp OR 'data mining'/exp OR 'automated diagnosis') AND ('prenatal diagnosis'/exp OR 'prenatal screening'/exp OR 'fetus echography'/exp OR 'obstetric ultrasonography' OR 'prenatal ultrasound' OR 'detailed anatomy ultrasound' OR 'anatomy scan' OR 'nuclear magnetic resonance imaging'/exp OR 'three-dimensional imaging'/exp) AND ('cost benefit analysis'/exp OR 'cost effectiveness analysis'/exp OR 'cost utility analysis'/exp OR 'economic evaluation'/exp)

Found papers = 3

Search excluding intervention: ('pregnant woman'/exp OR 'pregnant women' OR 'heart defects congenital diagnosis'/exp OR 'congenital heart disease'/exp OR 'fetus heart'/exp OR 'congenital heart malformation'/exp) AND ('prenatal diagnosis'/exp OR 'prenatal screening'/exp OR 'fetus echography'/exp OR 'obstetric ultrasonography' OR 'prenatal ultrasound' OR 'detailed anatomy ultrasound' OR 'anatomy scan' OR 'nuclear magnetic resonance imaging'/exp OR 'three-dimensional imaging'/exp) AND ('cost benefit analysis'/exp OR 'cost effectiveness analysis'/exp OR 'cost utility analysis'/exp OR 'economic evaluation'/exp)

Found papers = 467

OVID (Search Date: June 13, 2021)

Search including intervention: (Pregnant Women/ or Heart Defects, Congenital/ or Fetal Heart) AND (Machine Learning/ or Artificial Intelligence/ or Diagnosis, Computer-Assisted/ or Data Mining) AND (prenatal diagnosis/ or Ultrasonography, Prenatal/ or Ultrasonography/ or Magnetic Resonance Imaging/ or Imaging, Three-Dimensional) AND (Cost-Benefit Analysis/ or Cost-Effectiveness Analysis/ or Cost-Utility Analysis/ or economic evaluation.mp)

Found papers = 0

Search excluding intervention: (Pregnant Women/ or Heart Defects, Congenital/ or Fetal Heart) AND (prenatal diagnosis/ or Ultrasonography, Prenatal/ or Ultrasonography/ or Magnetic Resonance Imaging/ or Imaging, Three-Dimensional) AND (Cost-Benefit Analysis/ or Cost-Effectiveness Analysis/ or Cost-Utility Analysis/ or economic evaluation.mp)

Found papers = 46

INAHTA (Search Date: June 13, 2021)

Search including intervention: (Pregnant Women OR Heart Defects, Congenital OR Fetal Heart) AND (machine learning OR artificial intelligence OR data mining) AND (prenatal diagnosis OR Ultrasonography, Prenatal OR Ultrasonography OR Magnetic Resonance Imaging OR Imaging, Three-Dimensional) AND (Cost-Benefit Analysis OR Cost-Effectiveness Analysis OR Cost-Utility Analysis OR economic evaluation)

Found papers = 46

Search excluding intervention: (Pregnant Women OR Heart Defects, Congenital OR Fetal Heart) AND (prenatal diagnosis OR Ultrasonography, Prenatal OR Ultrasonography OR Magnetic Resonance Imaging OR Imaging, Three-Dimensional) AND (Cost-Benefit Analysis OR Cost-Effectiveness Analysis OR Cost-Utility Analysis OR economic evaluation)

Found papers = 125

WILEY (Search Date: June 15, 2021)

Search including intervention: "pregnant woman OR congenital heart defects OR fetal heart OR congenital heart malformation" anywhere and machine learning" OR "artificial intelligence" OR "data mining"" anywhere and "prenatal diagnosis OR fetus echography OR prenatal Ultrasonography OR Obstetric ultrasonography OR Prenatal ultrasound OR Magnetic Resonance Imaging" anywhere and "cost-benefit analysis OR economic evaluation OR Cost-Effectiveness Analysis OR Cost-Utility Analysis" anywhere

Found papers = 163

Search excluding intervention: "pregnant woman OR congenital heart defects OR fetal heart OR congenital heart malformation" anywhere and "prenatal diagnosis OR fetus echography OR prenatal Ultrasonography OR Obstetric ultrasonography OR Prenatal ultrasound OR Magnetic Resonance Imaging" anywhere and "cost-benefit analysis OR economic evaluation OR Cost-Effectiveness Analysis OR Cost-Utility Analysis" anywhere

Found papers = 1525
